# Sulbactam–Durlobactam in the Treatment of Multidrug-Resistant *Acinetobacter baumannii:* A Narrative Review

**DOI:** 10.3390/antibiotics15050499

**Published:** 2026-05-16

**Authors:** Szymon Viscardi, Patrycja Lipska, Piotr Niezgódka, Anna Duda-Madej

**Affiliations:** 1 Faculty of Medicine, Wroclaw Medical University, Ludwika Pasteura 1, 50-367 Wrocław, Poland; szymon.viscardi@student.umw.edu.pl (S.V.); patrycja.lipska@student.umw.edu.pl (P.L.); piotr.niezgodka@student.umw.edu.pl (P.N.); 2 Department of Microbiology, Faculty of Medicine, Wroclaw Medical University, Chałubińskiego 4, 50-368 Wrocław, Poland

**Keywords:** *Acinetobacter baumannii*, antimicrobial resistance (AMR), β-lactamase inhibitor, durlobactam, ETX2514, multidrug resistance (MDR), sulbactam

## Abstract

The increasing prevalence of infections caused by multidrug-resistant (MDR) Gram-negative bacteria represents a major global public health challenge. Among hospital-acquired infections (HAIs), ventilator-associated pneumonia (VAP) caused by non-fermenting Gram-negative pathogens, particularly the *Acinetobacter baumannii-calcoaceticus* complex, it is associated with limited therapeutic options and high mortality. Sulbactam–durlobactam is a novel combination consisting of sulbactam, a β-lactamase inhibitor with intrinsic activity against *Acinetobacter* spp., and durlobactam, a diazabicyclooctane β-lactamase inhibitor targeting Ambler class A, C, and D enzymes. This review summarizes current evidence on the pharmacological properties, clinical efficacy, and resistance mechanisms associated with this combination. Clinical trials have demonstrated that sulbactam–durlobactam is non-inferior to colistin in the treatment of infections caused by carbapenem-resistant *A. baumannii*, with a significantly lower risk of nephrotoxicity. The combination is generally well tolerated and represents a promising therapeutic option for difficult-to-treat infections. However, emerging resistance mechanisms, including PBP3 mutations, metallo-β-lactamase production, and efflux pump overexpression, may limit its long-term effectiveness. Further research is required to better understand resistance development and optimize clinical use.

## 1. Introduction

The increasing incidence of infections caused by multidrug-resistant (MDR) Gram-negative bacteria (GNB) is a major clinical and epidemiological challenge. This problem affects both countries with varying levels of access to healthcare systems. Thus, this issue is global in nature and is characterized by an alarmingly rapid increase. According to epidemiological data, GNB account for 49.7–95.3% of pneumonia cases worldwide [1]. Their prevalence is particularly high in cases of hospital-acquired pneumonia (HAP), especially those associated with ventilator-associated pneumonia (VAP) [2,3]. These infections contribute to prolonged hospital stays and increased risk of death. Pathogens with an increasing prevalence of MDR strains include *Acinetobacter baumannii*, *Pseudomonas aeruginosa*, and *Klebsiella pneumoniae* [4]. Their tendency to rapidly acquire resistance mechanisms limits the effectiveness of standard antibiotic therapy. An example of a dominant pathogen responsible for as many as 79.9–95% of lower respiratory tract infections is XDR (extensively drug-resistant)/MDR strains of *A. baumannii* [5,6], which are also responsible for as many as 40–75% of fatal cases, depending on the country [7,8,9,10]. This high percentage is primarily associated with resistance to classes of antibiotics, namely carbapenems and fluoroquinolones, which are routinely used in the treatment of such infections [11,12,13].

Antibiotic resistance in GNB is a multifactorial and dynamic phenomenon. The mechanisms leading to an increase in MDR and XDR phenotypes involve: (i) a decrease in outer membrane permeability (e.g., mutations in genes encoding porins, such as OmpK35/OmpK36 in *K. pneumoniae* [14,15] and OprD in *P. aeruginosa* [16]); (ii) overexpression of efflux pumps (e.g., AdeABC in *A. baumannii* [17,18], MexAB-OprM in *P. aeruginosa* [19,20]); (iii) modification of the antibiotic target site (e.g., mutations in genes encoding PBPs, such as PBP3 in *A. baumannii*) [21,22]; and (iv) the ability to form a biofilm, a physical and metabolic barrier to antibiotic penetration [23]. However, a key element of this complex bacterial defense system against antimicrobial drugs is the ability to produce enzymes, such as β-lactamases. In MDR and XDR phenotypes, these mechanisms often act in concert, creating complex resistance profiles that limit the effectiveness of available therapies [24,25,26].

Therefore, new therapeutic strategies should address multiple resistance mechanisms [27]. This remains a key challenge in the treatment of infections caused by GNB. The response to this growing resistance is a therapeutic strategy involving the combination of available antimicrobial agents with new ones. The most common combinations are those of antibiotics with enzyme inhibitors [28]. This approach, which leverages a well-established pathophysiological mechanism, is highly effective in treating difficult-to-treat infections.

Among the mechanisms mentioned, β-lactamases are of particular clinical significance, as their production inactivates β-lactam antibiotics [29]. This is one of the most important resistance mechanisms in GNB, as it excludes from therapy a vast group of antibiotics with a relatively low potential for causing side effects [30,31,32]. The combinations developed to date, namely ceftazidime–avibactam [33], meropenem–vaborbactam [34,35], and imipenem–relebactam [36,37], have restored the activity of antibiotics in this group against strains producing class A, C, and D β-lactamases [38,39,40]. The complementary mechanisms of action of the antibiotic and the inhibitor result in a significant increase in treatment efficacy, which is particularly important in the case of life-threatening infections.

Among the new therapies, the combination of two β-lactamase inhibitors, sulbactam and durlobactam (SBT-DBT), is particularly noteworthy for its unique activity against *Acinetobacter* spp. [41,42]. Unlike other inhibitors of these enzymes, sulbactam (SBT) exhibits direct antibacterial activity with a mechanism of action identical to that of β-lactam antibiotics [43]. Durlobactam (DBT), in turn, effectively inhibits β-lactamases of classes A, C, and D, including OXA-type carbapenemases [44]. This combination appears to be a promising therapeutic strategy, as it targets key resistance mechanisms and may reduce the risk of rapid selection of resistant strains [45]. Despite the increasing amount of data, the currently available information comes from diverse sources, including in vitro studies, pharmacological analyses, and limited clinical trials, highlighting the need for a comprehensive review.

This article aims to compile current data on the pharmacological properties, clinical efficacy, and resistance mechanisms associated with the use of SBT-DBT compared to other available combination therapies. Particular attention was devoted to the analysis of epidemiological data in the context of treating infections caused by carbapenem-resistant strains of *A. baumannii*. 

## 2. Characteristics of Sulbactam-Durlobactam

### 2.1. Chemical Structure of Sulbactam

**Figure 1:**
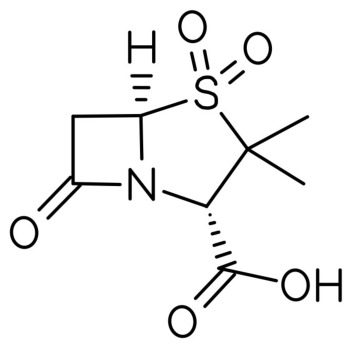
Molecular structure of sulbactam (two-dimensional).

SBT, with the molecular formula (2*S*,5*R*)-3,3-dimethyl-4,4,7-trioxo-4λ^6^-thia-1-azabicyclo[3.2.0]heptane-2-carboxylic acid (Figure 1, is a β-lactamase inhibitor (BLI) and an analog of the fundamental penam scaffold [46]. SBT, similar to clavulanic acid and tazobactam, possesses properties that protect the β-lactam ring and expand the antibacterial spectrum of β-lactam antibiotics [47]. Consequently, SBT is often combined with other antibacterial agents. For instance, the ampicillin/sulbactam (SAM) combination is used in the treatment of intra-abdominal conditions, skin disorders, and gynecological diseases [48]. SBT is an inhibitor of type A β-lactamases, exhibiting antimicrobial activity against specific bacterial species such as *Acinetobacter* spp., *Neisseria gonorrhoeae*, and *Bacteroides fragilis*. The activity of SBT against *A. baumannii* involves the inhibition of penicillin-binding proteins (PBPs)—PBP1 and PBP3—and varies depending on the bacterial strain and resistance level [43].

### 2.2. Chemical Structure of Durlobactam

**Figure 2:**
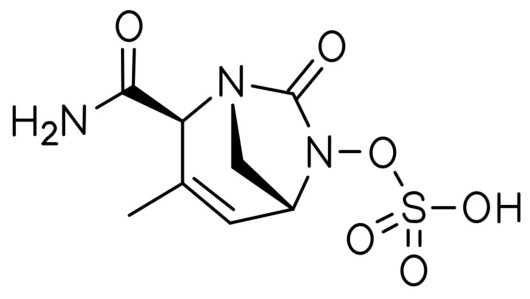
Molecular structure of durlobactam (two-dimensional).

Durlobactam (DBT, ETX2514), with the molecular formula [(2*S*,5*R*)-2-carbamoyl-3-methyl-7-oxo-1,6-diazabicyclo[3.2.1]oct-3-en-6-yl] hydrogen sulfate (Figure 2), is a diazabicyclooctane (DBO) non-β-lactam BLI [49]. It exhibits potent activity against a broad spectrum of class A, C, and D serine β-lactamases, as classified by the Ambler system [41,50]. DBT represents a novel advancement among β-lactamase inhibitors currently available on the market due to its expanded spectrum of activity. Specifically, unlike other DBO BLIs (avibactam, zidebactam, nacubactam, or relebactam), it effectively targets the widely distributed class D carbapenemases (OXA enzymes) in *A. baumannii* [41]. ETX2514 was designed based on avibactam by incorporating a methyl group at the C3 position and introducing a double bond between C3 and C4. These modifications resulted in improved inhibitor performance in terms of chemical activity and β-lactamase binding [51,52].

### 2.3. Combination of Sulbactam and Durlobactam

SBT-DBT is a combination of two active compounds, marketed under the trade name XACDURO^®^, formulated by Entasis Therapeutics Inc. The product is intended for the treatment of infectious diseases caused by the *Acinetobacter baumannii-calcoaceticus* complex (ABC). This specific drug combination, through the inclusion of DBT, prevents the degradation of SBT by β-lactamases produced by ABC [50]. SBT-DBT was approved by the United States Food and Drug Administration (FDA) in 2023 for the management of patients aged 18 years or older. It is registered for the treatment of hospital-acquired bacterial pneumonia (HABP) and ventilator-associated bacterial pneumonia (VABP) caused by the *Acinetobacter baumannii-calcoaceticus* complex. Given the growing need for additional treatment options and the significant public health threat posed by *Acinetobacter* bacteria, SBT-DBT should be used exclusively for infections caused by susceptible ABC strains [53]. 

### 2.4. Pharmacokinetic Properties

Both compounds are primarily eliminated via the renal route. Consequently, in 2019, O’Donnell et al. investigated the tolerance, pharmacokinetics, and safety of SBT-DBT in individuals with various degrees of renal impairment compared to healthy subjects. The mean concentrations of SBT and DBT increased as renal function declined in healthy individuals, those with renal impairment (RI), and patients with end-stage renal disease (ESRD). Regarding the median T_max_, it was approximately 3 h for both ETX2514 (DBT) and SBT across all treatment groups. DBT and SBT differed in their half-lives: the half-life ranged from 2.3 to 7.1 h for DBT, and from 1.8 to 10.0 h for SBT. In both cases, the half-life increased with the severity of renal impairment. The majority of ETX2514 and SBT elimination occurred within 24 h post-administration. Furthermore, hemodialysis effectively removed both compounds from plasma, which is clinically relevant for ESRD patients [54]. Moreover, SBT-DBT undergoes minimal metabolism in the body, with SBT exhibiting 38% plasma protein binding, while DBT binds to plasma proteins at a rate of 10% [55]. These findings support dose adjustment in renal impairment and confirm predictable pharmacokinetics.

## 3. Clinical Application

### 3.1. Dosage

SBT-DBT is administered at a fixed dose of 2 g (1 g SBT/1 g DBT) across all renal function categories, with dosing frequency adjustments as necessary to optimize and simplify administration. For instance, XACDURO is administered every 6 h as a 3 h infusion in patients with a creatinine clearance (CLcr) between 45 and 129 mL/min. In contrast, for patients with a CLcr of ≥130 mL/min, an increased dosing frequency of every 4 h is recommended. Conversely, patients with a lower CLcr ranging from 30 to 44 mL/min require extended dosing intervals of every 8 h. The treatment duration may extend up to 14 days [55,56,57]. The conducted pharmacodynamic and pharmacokinetic analyses support the proposed dose of 2 g (1 g SBT/1 g DBT) for the management of patients with *A. baumannii* isolates [58]. Interestingly, XACDURO is approved for use in breastfeeding women. SBT reaches low levels in maternal milk, which is unlikely to have a negative impact on nursing infants. There are no available data on the transfer of DBT into breast milk; however, it is estimated that it likely attains levels similar to those of SBT [59].

### 3.2. Indications

According to the FDA’s guidelines, SBT-DBT is recommended for the treatment of hospital-acquired bacterial pneumonia (HABP) and ventilator-associated bacterial pneumonia (VABP) caused by the *Acinetobacter baumannii-calcoaceticus* (ABC) complex [53]. On the other hand, the European Medicines Agency (EMA) has not yet published official guidelines regarding the use of the SBT-DBT combination. The only available information pertains to the approval of an amendment to the agreed Pediatric Investigation Plan for durlobactam/sulbactam.

### 3.3. Adverse Effects

SBT-DBT is generally described as well-tolerated. The most frequently reported adverse effects included headaches, nausea, and injection site phlebitis [60]. Among the patients studied by O’Donnell, unfavorable outcomes were observed in 6 out of 34 individuals receiving SBT-DBT. These included dizziness, epistaxis, falls, infusion site extravasation, foot fractures, mucosal dryness, nausea, and viral upper respiratory infections [54]. No drug–drug interactions were observed between DBT, SBT, imipenem, and cilastatin in a clinical study conducted in healthy subjects [55]. Overall, the safety profile appears favorable compared to polymyxins.

## 4. Antimicrobial Activity of Sulbactam-Durlobactam

### 4.1. Clinical Trials (Phase 1–3)

The phase 3 ATTACK trial was a multicenter, randomized, active-controlled non-inferiority study comparing SBT-DBT with colistin (COL) for the treatment of infections caused by the *Acinetobacter baumannii–calcoaceticus* complex [56]. Patients were randomized in a 1:1 ratio, and an additional observational cohort included individuals in whom COL could not be used. The most common infections in the randomized cohort were HAP and ventilator-associated bacterial pneumonia (VABP). In the SBT-DBT and COL groups, HAP accounted for 38% and 59% of cases, respectively, while VABP accounted for 48% and 47%. In the observational cohort, bloodstream infections (61%) and VABP (25%) were predominant. The ATTACK clinical trial endpoints and treatment regimens used are shown in Table 1.

Among patients included in the microbiologically modified intention-to-treat (m-mITT) population, a high proportion of infections were caused by carbapenem-resistant *A. baumannii* (CRAB) (63/77 in the SBT-DBT group and 62/78 in the COL group). Overall, 96% of isolates were carbapenem-resistant and classified as MDR, and 95% were considered difficult-to-treat. SBT-DBT demonstrated non-inferior efficacy compared to COL, with similar rates of treatment-emergent adverse events (88% vs. 94%) and a significantly lower incidence of nephrotoxicity (13% vs. 38%) [56]. In the mash, clinical results also showed a favorable SBT-DBT profile when compared to colistin, including: response at the end of therapy (86% vs. 61%, respectively), response in the cure test (68% vs. 42%). These findings support its use as a safer alternative to COL-based regimens, particularly in severe infections such as ventilator-associated pneumonia. A randomized controlled trial by Sagan et al. revealed that SBT-DBT in combination with IMI/CIL showed a favorable pharmacokinetic and safety profile compared to placebo. It has been proven that the preparation in the treatment of cUTIs (complicated urinary tract infection, including acute pyelonephritis) led to an overall rate of success comparable to a group of patients obtaining a placebo (76.6% vs. 81%—in m-mITT population). A comparable overall rate of success was obtained in microbiologically evaluable analysis (80% vs. 81%). Significantly, patients from the placebo group (treated with baseline IMI/CIL regimen) and SBT-DBT-treated cohort, who were diagnosed with IMI-NS *Enterobacteriaceae* infection, achieved 86% clinical success in the test of cure after administration of the SBT-DBT regimen [61].

### 4.2. In Vitro Studies

SBT-DBT shows consistently high activity against clinical isolates of *Acinetobacter baumannii-calcoaceticus* complex (ABC-C), MDR, XDR, and CRAB strains. In studies involving isolates from different geographical regions and periods (2016–2024), the effectiveness of the preparation (MIC ≤ 4 μg/mL) usually exceeded 95%, reaching values up to 98–99% [62,63,64]. Importantly, high activity was observed despite the low sensitivity of the studied populations to carbapenems (MER, IMI), often below 50% [62]. The preparation also induced 100% sensitization of CRAB to COL and aminoglycoside antibiotics [65]. Analysis of MIC values indicates that SBT-DBT achieves MIC90 ≤ 4 μg/mL for the vast majority of isolates (approx. 97–98%), and efficacy is also maintained against strains resistant to numerous classes of antibiotics, including COL, MIN, ACN, and CFP [62,65,66,67]. For CRAB isolates, the susceptibility rate was typically 88–92%, with an MIC50/90 in the range of 0.5–4 μg/mL [68,69]. Additionally, significant activity was demonstrated against cefiderocol-non-susceptible (CFD-NS) strains (approx. 66–73%) [66,70]. A summary of the in vitro activity of SBT-DBT and its drug combinations against *A. baumannii* and other GNB is presented in Table 2.

However, sensitivity to SBT-DBT depends on the profile of resistance mechanisms. The preparation shows very high activity against strains producing OXA-type carbapenemases (especially OXA-40 and OXA-58—up to 100% sensitivity), while significantly lower efficacy, especially against strains producing MBL, where in single analyses the activity was limited (e.g., ~20% for NDM-positive isolates) [70,76]. Mutations in the PBP3 protein also remain a key mechanism limiting efficacy [64]. An important feature of SBT-DBT is its ability to act synergistically with other antibiotics. Numerous studies have shown that the addition of DBT leads to a significant reduction in MIC values for carbapenems and other β-lactams [72,77]. The combination with IMI shows particularly high efficacy, outperforming monotherapy against both COL-NS and CFD-NS strains (approx. 96% for combined vs. 85–88% for SBT-DBT alone) [78]. Strong synergistic effects (FICI ≤ 0.5) were also observed with many antibiotics, including CFP (cefepime), CAZ-AVI, MER, IMI, MIN, and ACN, as well as a significant reduction in bacterial load in in vitro models [73,79]. Despite comparable or lower efficacy against colistin in some analyses (e.g., 71% vs. 91%), the potential use of SBT-DBT is particularly important due to its more favorable safety profile and lower risk of nephrotoxicity [63,76]. Current data also indicate the advantage of combination therapies (especially with IMI) and the higher efficacy of the SBT-DBT-based regimens compared to other β-lactamase inhibitors (e.g., AVI) [65,71,75]. Taken together, the available data indicate that SBT-DBT appears to be a promising therapeutic option for the treatment of *A. baumannii* infections, particularly in highly resistant populations, although its efficacy may be limited in strains producing MBL and harboring PBP3 mutations. The summary of total percentage susceptibility of tested isolates to SBT-DBT is presented in Table 3.

Taken together, these data indicate that SBT-DBT maintains consistent in vitro activity (>90–95%) across diverse geographic regions and resistance phenotypes, although reduced efficacy is observed in MBL-producing isolates.

### 4.3. Case Reports

Several case reports highlight the potential role of SBT-DBT as salvage therapy in severe infections caused by CRAB, particularly in cases with limited treatment options. Tiseo et al. described successful treatment of VABP caused by COL- and CFD-resistant CRAB harboring multiple resistance determinants, including class D β-lactamases, as well as mutations in PBP3 and siderophore receptor genes. Despite extensive resistance, the isolate remained susceptible to SBT-DBT (MIC = 1.5 µg/mL), and targeted therapy resulted in 30-day survival [80]. Similarly, Van Natta et al. reported successful treatment of VABP using a combination regimen including SBT-DBT, following failure of multiple prior therapies. Clinical improvement and microbiological eradication were achieved despite the presence of multiple resistance mechanisms, including β-lactamases and efflux pump overexpression [81]. Additional reports support these findings. Snowdin et al. described successful treatment of *A. baumannii* neuroinfection following replacement of COL due to nephrotoxicity, while Xiong et al. demonstrated efficacy in a post-transplant patient with pneumonia and sepsis of CRAB etiology, resistant to multiple antibiotic classes [82,83]. Overall, available clinical reports suggest that SBT-DBT may represent an effective salvage therapy option in severe MDR infections, particularly when standard treatment regimens fail or are not tolerated. However, these findings should be interpreted with caution due to heterogeneity among included studies.

## 5. Resistance to Sulbactam-Durlobactam

### 5.1. β-Lactamases Play a Role in Resistance to SBT-DBT

β-Lactamase production represents the key mechanism limiting the activity of SBT-DBT, particularly in CRAB isolates. While DBT effectively inhibits Ambler’s class A, C, and D enzymes (serine enzymes), resistance may still arise due to enzymes not covered by its inhibitory spectrum (class B—metallo-β-lactamases) or due to high-level expression of β-lactamases [79]. The ability of SBT-DBT to overcome bacterial resistance mediated via β-lactamases is presented on Table 4.

Summarized data indicate that SBT-DBT retains activity against most serine β-lactamases, including OXA-type carbapenemases commonly found in *A. baumannii*. However, the lack of activity against MBL (e.g., NDM) represents a major limitation, as these enzymes are increasingly reported in clinical CRAB isolates. Literature data indicate that resistance to SBT-DBT is strongly associated with the production of multiple β-lactamases in *A. baumannii*, particularly class C (ADC) and class D (OXA-type) enzymes, which are intrinsic and commonly overexpressed in CRAB isolates. Studies consistently demonstrate that enzymes such as OXA-23, OXA-24, and OXA-51, often coexisting with ADC variants, contribute to reduced susceptibility to SBT-DBT, especially when expressed at high levels [66,68,76,84]. Importantly, resistance is not driven by a single enzyme but rather by the cumulative effect of multiple β-lactamases. The co-expression of class A enzymes (e.g., TEM-1) alongside class C and D enzymes further enhances resistance, suggesting a synergistic effect between different enzymatic systems [84,85]. A critical limitation of SBT-DBT therapy arises in the presence of MBLs, such as NDM-1, NDM-5, which are not inhibited by DBT. Their presence significantly reduces the efficacy of the combination, even when serine BLs are effectively inhibited. In such cases, combination strategies, including aztreonam-based regimens, have been proposed to overcome resistance [62,85]. Taken together, these findings indicate that resistance to SBT-DBT is a multifactorial phenomenon driven primarily by the accumulation and overexpression of β-lactamases, with MBLs representing the most clinically significant threat due to the lack of effective inhibition.

### 5.2. Mutation of the PBP3 Protein

Mutations in penicillin-binding protein 3 (PBP3), a key target of SBT, represent a primary non-enzymatic mechanism of resistance to SBT-DBT. These alterations reduce the binding affinity of SBT to its target, thereby diminishing its antibacterial activity. Current evidence indicates that not all PBP3 mutations contribute equally to resistance. Among the most clinically relevant substitutions, T526S has been consistently associated with a significant increase in MIC values (up to 8-fold), indicating a strong impact on SBT-DBT susceptibility. In contrast, the frequently reported A515V substitution appears to have a more limited effect when present alone, suggesting that it may act as a secondary (e.g., in the presence of OXA-24 expression) or facilitating mutation rather than a primary driver of resistance [62,69,76,84,85]. Additional mutations, including S390T, V505L, H370Y, and G523V, have also been identified in resistant isolates, with the latter being associated with a modest (~2-fold) increase in MIC for both SBT and SBT-DBT. These substitutions are thought to induce conformational changes that impair SBT binding, although their individual contributions to resistance vary and may depend on the genetic background of the strain [81,86]. Importantly, the accumulation of multiple PBP3 mutations appears to have an additive effect on resistance, particularly when combined with other mechanisms such as β-lactamases overexpression. Longitudinal studies further suggest that these mutations may emerge during therapy under selective pressure of β-lactam antibiotics targeting PBPs [67,81]. Exposure to different β-lactam antibiotics has been shown to select for PBP3 mutations (e.g., A515V, H370Y), contributing to the development of resistance to both SBT-DBT and CFD [81]. Notably, PBP3 alterations do not universally confer cross-resistance—to other β-lactams. For instance, some mutations associated with CFD resistance were not linked to reduced susceptibility to SBT-DBT, indicating that the structural determinants of resistance differ between these agents [66]. Overall, PBP3 mutations represent a critical and evolving mechanism of resistance to SBT-DBT, with certain substitutions (particularly T526S) having the greatest clinical relevance due to their pronounced impact on antimicrobial susceptibility.

### 5.3. Efflux-Mediated Resistance to SBT-DBT

Efflux-mediated resistance represents an important non-enzymatic mechanism contributing to reduced susceptibility to SBT–DBT in *A. baumannii*. Among the various efflux systems, resistance–nodulation–division (RND) pumps, particularly AdeABC, AdeFGH, and AdeIJK, play a central role in MDR through active extrusion of antibiotics [45,85,87]. Current evidence suggests that overexpression of these efflux systems leads to decreased intracellular concentrations of SBT-DBT, thereby reducing its antibacterial activity. This phenomenon is frequently observed in combination with other resistance mechanisms, such as β-lactamase overproduction and PBP3 mutations, making it difficult to assess the individual contribution of efflux pumps in clinical isolates [67,68]. However, it remains unclear to what extent mutations affecting regulatory systems (e.g., AdeRS, AdeL, and AdeN), which control the expression of AdeABC, AdeFGH, and AdeIJK, directly contribute to SBT-DBT resistance in MDR *A. baumannii* isolates. [85]. In addition to RND systems, other efflux transporters (e.g., AbeS, AbaQ, and AbaR) and tetracycline-associated pumps such as TetA(41) have also been identified in resistant strains, further supporting the role of efflux in MDR phenotypes [68]. Importantly, evidence from individual clinical cases suggests that efflux-mediated resistance may occur independently of other major mechanisms. For instance, a missense mutation in the *adeJ* gene was associated with reduced susceptibility to SBT-DBT in the absence of metallo-β-lactamase production and PBP3 mutations, highlighting the potential of efflux pumps as a standalone driver of resistance [85]. AdeJ variant was also identified as a potential factor in SBT-DBT resistance in CRAB isolates [81]. Overall, efflux pump overexpression represents a significant contributor to SBT-DBT resistance, particularly as part of a multifactorial resistance phenotype, although its isolated impact requires further investigation.

### 5.4. Spread of Resistance to SBT-DBT by a Mobile Genome

**Figure 3:**
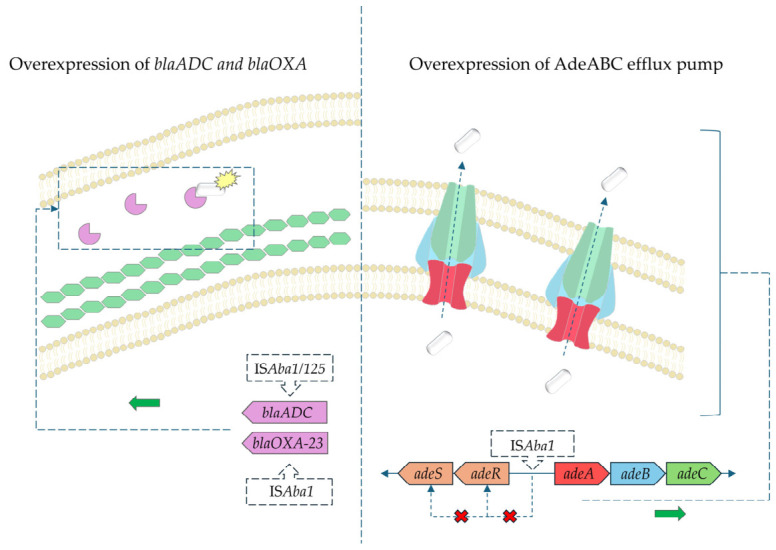
Estimated molecular ways of SBT-DBT resistance among *A. baumannii* associated with insertion sequences IS*Aba1* and IS*Aba125*.

Mobile genetic elements (MGEs) play a critical role in the dissemination and regulation of antimicrobial resistance determinants in *A. baumannii*, including those affecting susceptibility to SBT-DBT and other antibiotics [67,88]. Insertion sequences such as ISAba1 and ISAba125 are of particular importance, as they can act as promoter elements that enhance the expression of β-lactamase genes when located upstream, including *blaADC, blaOXA-23, blaOXA-51, blaOXA-58,* and *blaNDM-1*. This leads to overexpression of these enzymes, increased β-lactam hydrolysis, and consequently elevated MIC values, potentially reducing the efficacy of SBT-DBT [68,76,89]. ISAba125 has also been shown to provide promoter sequences driving the expression of β-lactamase genes, including *blaNDM-1* [90,91]. MGEs also contribute to resistance indirectly by modulating efflux pump expression. For instance, insertion of ISAba1 within regulatory regions such as *adeRS* has been associated with overexpression of the AdeABC efflux system, leading to decreased susceptibility to multiple antibiotics [92,93]. Given the role of RND efflux pumps in reducing intracellular concentrations of SBT-DBT, such regulatory disruptions may further compromise treatment efficacy [67,68]. The summary of the possible impact of IS*Aba1/125* on development of SBT-DBT resistance among *A. baumannii* is depicted on Figure 3.

In addition to regulatory effects, MGEs facilitate horizontal gene transfer of resistance determinants. Transposon Tn125, typically associated with *blaNDM-1*, has been identified in both chromosomal and plasmid contexts. In plasmids such as IncX3, *blaNDM-1* is often found within truncated or remnant forms of Tn125, reflecting its role in gene mobilization. These elements are often located on conjugative plasmids (e.g., IncX3), enabling rapid dissemination across bacterial populations and promoting extensively drug-resistant phenotypes [94,95,96]. Overall, MGEs contribute to SBT-DBT resistance through a combination of gene mobilization, transcriptional upregulation of resistance determinants, and indirect modulation of efflux systems, highlighting their central role in the evolution and spread of MDR in *A. baumannii*.

## 6. Material and Methods

A literature search was conducted using PubMed, Scopus, and Web of Science databases up to March 2026. Keywords included ‘sulbactam’, ‘durlobactam’, ‘ETX2514’, and ‘*Acinetobacter baumannii’*. Only English-language articles were included. Clinical trials, in vitro studies, and relevant reviews were considered. Studies were selected based on relevance, recency, and methodological quality. A total of 96 articles were included in the final analysis. Table 1 summarizes the ATTACK clinical trial end-points. Table 2 stands for MIC values of SBT-DBT against selected clinical isolates tested in in vitro studies. Table 3 presents a summary of the percentage susceptibility of the tested *A. baumannii* isolates to SBT-DBT. Table 4 describes the ability of SBT-DBT to inhibit various β-lactamases according to Ambler. Table 5 summarizes the novel BL/BLI combinations. Figure 1 and Figure 2 present two-dimensional structures of SBT and DBT, respectively. Figure 3 depicts the scheme of IS*Aba1/125* sequences’ influence in development of SBT-DBT resistance among *A. baumannii*.

## 7. Conclusions

The increasing prevalence of antimicrobial resistance among GNB is a major global health challenge and continues to drive the development of novel therapeutic strategies. Within the β-lactam class, recent advances include the introduction of siderophore cephalosporins, such as cefiderocol, and next-generation β-lactamase inhibitors based on non-β-lactam scaffolds, including diazabicyclooctane and boronic acid derivatives. SBT-DBT represents a unique combination of a well-established agent with a novel β-lactamase inhibitor, demonstrating significant activity against the *Acinetobacter baumannii–calcoaceticus* complex. Clinical evidence, including the phase 3 ATTACK trial, indicates non-inferior efficacy compared to colistin, with a substantially improved safety profile, particularly in terms of nephrotoxicity. However, emerging resistance, particularly associated with PBP3 mutations and MBL production, remains a concern. Novel BL/BLI combinations represent one of the most important directions in the development of therapies against MDR GNB, particularly in response to the increasing prevalence of carbapenemase-producing pathogens. Currently investigated BL/BLI combinations aim to expand activity against metallo-β-lactamase-producing strains while providing more effective and accessible therapeutic options for the treatment of MDR/XDR infections (Table 5).

Nevertheless, SBT–DBT represents one of the most promising targeted therapies against CRAB infections currently available. Therefore, continued surveillance and optimization of therapeutic strategies remain essential. While further development of β-lactam-based therapies is expected, the growing complexity of resistance highlights the need for novel approaches targeting alternative bacterial pathways. However, the clinical utility of such strategies remains to be confirmed in well-designed in vivo studies.

## Figures and Tables

**Table 1:** Summary of ATTACK phase-3 trial end-points in randomized cohort based on Kaye et al. [56].

Categories	SBT-DBT- Based Regimen [%]	COL- Based Regimen [%]
**Treatment scheme**	SBT-DBT (1.0 + 1.0 g in 3 h infusion every 6 h) + IMI/CIL (1.0 + 1.0 g in 1 h infusion every 6 h)	COL (2.5 mg/kg in 0.5 h infusion every 12 h) + IMI/CIL (1.0 + 1.0 g in 1 h infusion every 6 h)
**Primary end-point**		
**28-DACM** (CRAB-C m-mITT)	19%	32.3%
**Secondary end-points**		
**28-DACM** (CRAB-C microbiologically evaluable analysis)	17%	36%
**28-DACM** (ITT)	21%	33%
**28-DACM** (m-mITT)	20%	33%
**14-DACM** (CRAB-C m-mITT)	6%	19%
**14-DACM** (m-mITT)	8%	20%

Abbreviations: 14/28-DACM—14/28-day all-cause mortality, CRAB-C m-mITT—microbiologically modified intention to treat population with confirmed carbapenem-resistant *A. baumannii—calcoaceticus* complex infection, IMI/CIL—imipenem/cilastatin, ITT—intention to treat population—all patients randomized to study drug treatment (SBT-DBT or COL), m-mITT—all patients who obtained any amount of study drugs and were confirmed to be infected with CRAB-C pathogen.

**Table 2:** The summary of in vitro antimicrobial activity of SBT-DBT and its combination with other drugs against GNB.

Pathogens	MICs Values	Drug Regimen	References
*A. baumannii*	MIC50/90 = 1/2 µg/mL	SBT-DBT	[62,67]
	MIC50/90 = 1/4 µg/mL		
*A. calcoaceticus* *A. nosocomialis*	MIC50/90 = 0.5/1 µg/mL		
*A. pittii*	MIC50/90 = 0.5/2 µg/mL		
CRAB (harboring *bla* _OXA-23_)	MIC50/90 = 1/2 µg/mL		[71]
CRAB (harboring *bla* _OXA-40_)	MIC50/90 = 1/1 µg/mL		
CRAB	MIC50/90 = 4/8 µg/mL MIC50/90 = 2/4 µg/mL	SBT-DBT SBT-DBT + IMI	[69]
	MIC50/90 = 4/4 µg/mL MIC50/90 = 2/4 µg/mL		
*A. baumannii* (TEM-1/KPC-2/ADC-30/OXA-23/OXA-24)	MIC = 0.5 µg/mL	SBT-DBT	[72]
CRAB	MIC50/90 = 1/4 µg/mL		
*A. baumannii* (including IMI-NS/SBT-NS/MIN-NS isolates)	MIC50/90 = 1/2 µg/mL	SBT-DBT	[63]
*A. baumannii* IMI-SC	MIC50/90 = 0.25/0.5 µg/mL		
*A. baumannii* (including IMI/SBT/MIN-NS isolates)	MIC50/90 = 1/2 µg/mL	SBT-DBT + IMI	
*A. baumannii* IMI-SC	MIC50/90 = 0.12/0.25 µg/mL		
*A. baumannii* (OXA-23/66/TEM-1/ADC-25,82,91,162)	MIC = 2–32 µg/mL	SBT-DBT	[73]
*E. coli* (e.g., KPC-3, OXA-48)	MIC ≤ 0.125 µg/mL		
*K. pneumoniae* (KPC-3, *oqxA,* CTX-M-15, NDM-1)			
*A. baumannii* OXA-23	MIC50/90 = 0.5/2 mg/L	SBT-DBT	[74]
*A. baumannii* OXA-24/40	MIC50/90 = 0.25/1 mg/L		
CRAB	MIC50/90 = 2/4 mg/L		
CRAB	MIC50/90 = 2/8 mg/L	SBT-DBT	[75]
	MIC50/90 = 16/>32 mg/L	SBT-AVI	
	MIC50/90 = 2/8 mg/L	SBT-DBT + MER	
	MIC50/90 = 1/4 mg/L	SBT-DBT + IMI	

Abbreviations: ADC—*Acinetobacter* derived cephalosporinase, *bla*—β-lactamase encoding gene, AVI—avibactam, CTX-M-15—Ambler’s class A extended spectrum β-lactamase, CRAB—carbapenem-resistant *A. baumannii*, IMI—imipenem, KPC—*Klebsiella pneumoniae* carbapenemase, MIC—minimal inhibitory concentration, MIN—minocycline, NDM—New Delhi metallo-β-lactamase, NS—non-susceptible, *oqxA*—gene encoding *K. pneumoniae* efflux pump subunit, OXA—Ambler’s class D β-lactamase, SBT-DBT—sulbactam-durlobactam, SC—susceptible, TEM—Ambler’s class A narrow spectrum β-lactamase.

**Table 3:** Total percentage sensitivity of the tested isolates to SBT-DBT.

Region	Bacteria Phenotype	% Susceptibility	References
Global	ABC-C	98.3	[62]
Global	MDR/XDR ABC-C	>95	[62,67]
Europe	*A. baumannii* (incl. CRAB)	~97 (93.8)	[66]
Global	ABC-C	~98	[67]
Italy	CRAB	92	[68]
Greece	CRAB	87.9	[69]
Global	*A. baumannii* (BL positive)	71	[76]
PRC	*A. baumannii*	97.9	[63]
Israel	CRAB	100	[65]
PRC	CRAB	>95	[64]

Abbreviations: ABC-C—*A. baumannii-calcoaceticus* complex, BL—beta-lactamase, CRAB—carbapenem resistant *A. baumannii*, MDR—multi-drug-resistant, XDR—extensively drug-resistant, PRC—People Republic of China.

**Table 4:** The summary of β-lactamases inhibitory properties of SBT-DBT.

Class According to Ambler	Effect of SBT-DBT	β-Lactamase Example
A	Active against various serine β-lactamases	Narrow spectrum, e.g., TEM-1 Extended spectrum, e.g., CTX-M
B	Lack of inhibition	NDM-1, VIM, IMP
C	Broad-spectrum inhibition	ADC, e.g., ADC-7, ADC-30, ADC-25, ADC-73
D	Broad-spectrum inhibition	OXA-20, OXA-23, OXA-24, OXA-48, OXA-51, OXA-58, OXA-66

Abbreviations: TEM—Plasmid-encoded β-lactamase in GNB; NDM—New Delhi metallo-β-lactamase; ADC—*Acinetobacter*-derived cephalosporinase; OXA—Oxacillinase enzymes found in *Acinetobacter* spp.; VIM—Verona integron-encoded metallo-β-lactamase, IMP—imipenemase type metallo-β-lactamase.

**Table 5:** The summary of the cutting edge, novel BL/BLI combinations.

Combination	Trial Phase	Inhibitor Type	Ambler’s Activity	Antimicrobial Targets
Cefepime/ Taniborbactam	III	Cyclic boronate	A–D	CRE, *P. aeruginosa* (including NDM, VIM)
Cefepime/ Zidebactam	III	DBO	A, C, D B (enhancing cefepime activity via binding to PBP2)	CRE, non-fermenters (including KPC, MBL, AmpC)
Meropenem/ Nacubactam	II/III	DBO	A, C, D B (enhancing meropenem activity via binding to PBP2)	Various G(-) bacteria including KPC, AmpC, OXA, and MBL producers
Ceftibuten/ Ledaborbactam (QPX7728)	II/III	Cyclic boronate	A, C, D	CRE (especially KPC producers)
Cefepime/ Xeruborbactam	I	Cyclic boronate	A–D	CRE, non-fermenters, e.g., MBL, OXA producers
Aztreonam/ Relebactam	Early research	DBO	A, C B (via aztreonam) D (limited activity)	CRE (especially MBL producers) Non-fermenters (e.g., MBL, KPC producers)

Abbreviations list: AmpC—class C β-lactamase, CRE—Carbapenem-resistant *Enterobacterales,* DBO—diazabicyclooctane, KPC—*Klebsiella pneumoniae* carbapenemase, MBL—metallo-β-lactamase, NDM—New Delhi MBL, OXA—class D β-lactamase, PBP—penicillin binding protein, VIM—Verona integron-encoded MBL.

## Data Availability

No new data were created or analyzed in this study.
